# *Leishmania* Antigenuria to Predict Initial Treatment Failure and Relapse in Visceral Leishmaniasis/HIV Coinfected Patients: An Exploratory Study Nested Within a Clinical Trial in Ethiopia

**DOI:** 10.3389/fcimb.2018.00094

**Published:** 2018-03-29

**Authors:** Johan van Griensven, Bewketu Mengesha, Tigist Mekonnen, Helina Fikre, Yegnasew Takele, Emebet Adem, Rezika Mohammed, Koert Ritmeijer, Florian Vogt, Wim Adriaensen, Ermias Diro

**Affiliations:** ^1^Department of Clinical Sciences, Institute of Tropical Medicine, Antwerp, Belgium; ^2^Department of Clinical Sciences, University of Gondar, Gondar, Ethiopia; ^3^Médecins Sans Frontières, Amsterdam, Netherlands

**Keywords:** HIV, visceral leishmaniasis, relapse, treatment failure, antigen test, urine, prediction

## Abstract

**Background:** Biomarkers predicting the risk of VL treatment failure and relapse in VL/HIV coinfected patients are needed. Nested within a two-site clinical trial in Ethiopia (2011–2015), we conducted an exploratory study to assess whether (1) levels of *Leishmania* antigenuria measured at VL diagnosis were associated with initial treatment failure and (2) levels of *Leishmania* antigenuria at the end of treatment (parasitologically-confirmed cure) were associated with subsequent relapse.

**Methods:** *Leishmania* antigenuria at VL diagnosis and cure was determined using KAtex urine antigen test and graded as negative (0), weak/moderate (grade 1+/2+) or strongly-positive (3+). Logistic regression and Kaplan-Meier methods were used to assess the association between antigenuria and (1) initial treatment failure, and (2) relapse over the 12 months after cure, respectively.

**Results:** The analysis to predict initial treatment failure included sixty-three coinfected adults [median age: 30 years interquartile range (IQR) 27–35], median CD4 count: 56 cells/μL (IQR 38–113). KAtex results at VL diagnosis were negative in 11 (17%), weak/moderate in 17 (27%) and strongly-positive in 35 (36%). Twenty (32%) patients had parasitologically-confirmed treatment failure, with a risk of failure of 9% (1/11) with KAtex-negative results, 0% (0/17) for KAtex 1+/2+ and 54% (19/35) for KAtex 3+ results. Compared to KAtex-negative patients, KAtex 3+ patients were at increased risk of treatment failure [odds ratio 11.9 (95% CI 1.4–103.0); *P*: 0.025].

Forty-four patients were included in the analysis to predict relapse [median age: 31 years (IQR 28–35), median CD4 count: 116 cells/μL (IQR 95–181)]. When achieving VL cure, KAtex results were negative in 19 (43%), weak/moderate (1+/2+) in 10 (23%), and strongly positive (3+) in 15 patients (34%). Over the subsequent 12 months, eight out of 44 patients (18%) relapsed. The predicted 1-year relapse risk was 6% for KAtex-negative results, 14% for KAtex 1+/2+ and 42% for KAtex 3+ results [hazard ratio of 2.2 (95% CI 0.1–34.9) for KAtex 1+/2+ and 9.8 (95% CI 1.8–82.1) for KAtex 3+, compared to KAtex negative patients; *P*: 0.03].

**Conclusion:** A simple field-deployable *Leishmania* urine antigen test can be used for risk stratification of initial treatment failure and VL relapse in HIV-patients. A dipstick-format would facilitate field implementation.

## Introduction

Visceral Leishmaniasis (VL) is a vector borne protozoan disease caused by species of the *Leishmania donovani* complex. The parasite predominantly infects reticuloendothelial cells (van Griensven and Diro, 2012). Every year, 200,000–400,000 new VL cases are estimated to occur within approximately 70 countries. In the Mediterranean region and South America, VL is caused by *L infantum*. In East Africa and the Indian subcontinent, *L donovani* is prevalent (van Griensven and Diro, 2012).

HIV infection is one of the main risk factors for VL, and the HIV epidemic caused the re-emergence of VL in the endemic South-European countries (Desjeux and Alvar, 2003). VL/HIV coinfection is now a major problem in some low resource settings. The highest burden globally is found in North-West Ethiopia, where around 20% of VL patients are HIV co-infected (Diro et al., 2014). Management of VL/HIV patients is complicated. Besides high mortality and poor response to anti-leishmanial treatment, these patients are at high risk of VL relapse even when apparent cure is parasitologically confirmed from spleen or bone marrow aspirates (Diro et al., 2014). There are, however, only few indicators at hand to identify those at highest risk of failure or relapse, such as a history of previous VL episodes or low CD4 counts at VL diagnosis (Cota et al., 2011). Other laboratory risk factors—or biomarkers—in particular markers of *Leishmania* infection, have hardly been explored in resource-constrained settings.

The KAtex urine antigen test detects *Leishmania* antigen, which is a direct marker of infection. Its value to predict initial treatment failure has not been assessed in HIV coinfected patients. Presence of urine antigen during follow-up of HIV patients was found predictive of VL relapse in areas where *L infantum* is present (Riera et al., 2004), but has not been explored in *L donovani* endemic areas. As this test is easy to use, non-invasive, and relatively cheap, it could be particularly relevant for resource-constrained settings to help identify those HIV patients at higher risk of treatment failure or VL relapse who might hence benefit from more potent or longer treatment and close clinical follow-up after treatment.

Nested within a clinical trial on secondary prophylaxis, we conducted an exploratory study to assess whether (1) the level of *Leishmania* antigenuria measured at the time of diagnosis was associated with initial treatment failure and (2) the level of *Leishmania* antigenuria measured with the KAtex assay at the time of parasitologically confirmed cure—end of treatment—was associated with subsequent relapse in VL/HIV co-infected patients.

## Methods

This laboratory study was nested within a clinical trial conducted between 2011 and 2015 in two VL treatment sites in North-West Ethiopia (Diro et al., 2015, 2017). We obtained approval of the trial protocol from the Ethiopian regulatory authority, the National Research Ethics Review Committee, the University of Gondar Institutional Review Board (IRB), the Ethics Review Board of Médecins sans Frontières, the IRB of the Institute of Tropical Medicine, Antwerp and the Ethics Committee of Antwerp University Hospital. All participants provided written informed consent. The protocol was registered at Clinicaltrials.gov (code NCT01360762).

**Figure 1:**
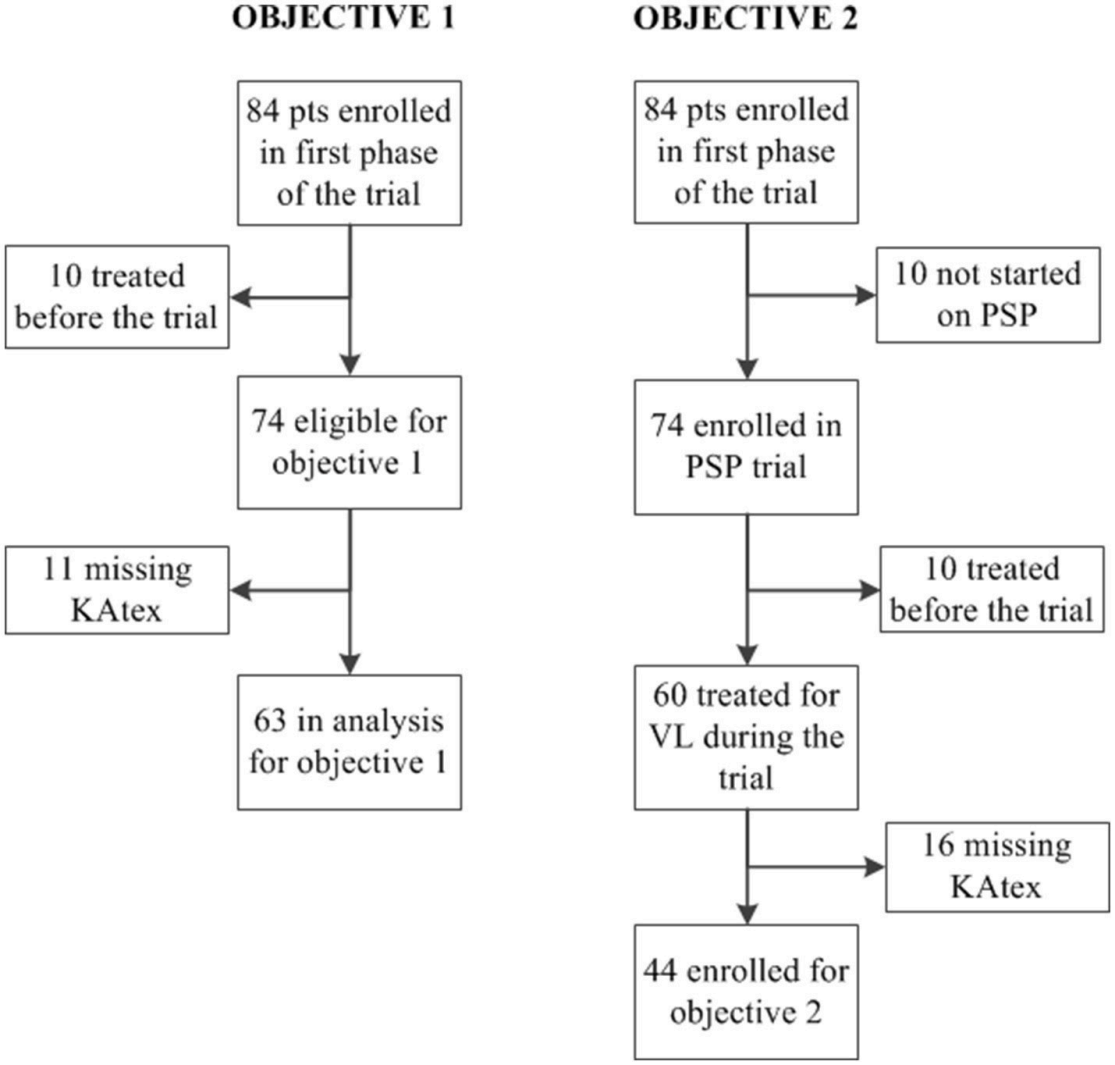
Flowchart displaying the number of patients included in analysis of objective 1 (association between urine antigen at visceral leishmaniasis (VL) diagnosis and the risk of treatment failure) and objective 2 (association between urine antigen at VL cure and the risk of relapse). PSP, pentamidine secondary prophylaxis.

The main objective of the trial was to determine the effectiveness, safety and feasibility of monthly administration of pentamidine as secondary prophylaxis to prevent VL relapse in 74 HIV-coinfected patients that had achieved microscopically confirmed parasitological cure. Secondary prophylaxis consisted of intravenous injections of 4 mg/kg of pentamidine isethionate (provided by Sanofi-Aventis) for a minimal period of 1 year. The main analysis focused on patient outcomes at 12 months after VL cure, and has been reported before. Pentamidine was found to be safe, effective and feasible to implement in resource-constrained settings (Diro et al., 2015). In this nested study, all patients recruited in the clinical trial with (1) KAtex test results at VL diagnosis and a test of cure result after the initial treatment (objective 1) or (2) KAtex results at the end of treatment (VL cure) and follow-up for relapse in the trial were included (objective 2). KAtex results were missing for some (11 missing for objective 1; 16 for objective 2), see Figure 1.

The KAtex test was done at the time of VL diagnosis and achieving VL cure, and was performed as per recommendations of the manufacturer (Kalon Biological Ltd., Guilford England). The KAtex urine assay is semi-quantitative, with three levels of agglutination: 1+: weakly positive; 2+: moderately positive; 3+: strongly positive. Agglutination of any degree visible to the naked eye was considered positive, whilst no agglutination was taken as negative. Urine samples were aliquoted within 3 h after collection and kept at −20°C, as KAtex tests were performed in batch on stored samples. The diagnosis of VL relied on parasite detection in tissue aspirates (spleen, bone marrow, or lymph node), and grading was done as reported before (WHO, 2010). Grading of the parasite load was as follows: 6+: >100 parasites/field; 5+: 10–100 parasites/field; 4+: 1–10 parasites/field; 3+: 1–10 parasites/10 fields; 2+: 1–10 parasites/100 fields; 1+: 1–10 parasites/1,000 fields; 0: no parasites/1,000 fields. Initial parasitological treatment failure was defined as the presence of tissue parasites at the end of the initial treatment. Initial cure was defined as the absence of tissue parasites at the end of the initial treatment, combined with clinical improvement. Treatment consisted of AmBisome 30–40 mg/kg (with or without miltefosine for 28 days) or sodium stibogluconate 20 mg/kg for 30 days or sodium stibogluconate 20 mg/kg and paromomycin 15 mg/kg for 17 days, as reported before (Diro et al., 2015, 2017). If not yet an antiretroviral treatment (ART) at VL diagnosis, this was started during hospitalization.

Associations between patient characteristics and the level of KAtex antigenuria was assessed using the Kruskal-Wallis test for continuous variables and the Fisher's exact test for binary/categorical variables. We calculated the proportion with initial parasitological treatment failure, stratified by KAtex result. We used logistic regression to quantify the association between the KAtex test result and the risk of initial treatment failure, with the strength of association expressed as odds ratio's (OR) and 95% confidence intervals (95% CI). KAtex 1+ and 2+ results were pooled together due to the low sample size in each of these categories. There were thus three categories: negative (0); weakly/moderately positive (1+/2+); strongly positive (3+). The 1 year risk of relapse was determined using Kaplan-Meier methods, stratified by KAtex result, with follow-up time starting at the time of achieving VL cure and censored at the time of death, relapse or lost to follow-up, or after 12 months if none of these events had occurred. We used hazard ratio's (HR) and 95% CIs to quantify the association between the KAtex test result and the risk of relapse. To assess whether the KAtex result could be a non-invasive measure of the tissue parasite load, we assessed the association between the KAtex level (non-invasive test) and the parasite grading on tissue aspiration (invasive test). The association between the tissue parasite grading and the risk of initial treatment failure and relapse was assessed as well. All analysis was done using Stata version 14.

## Results

Sixty-three coinfected adults were included in the analysis to assess the association between *Leishmania* antigenuria at VL diagnosis and initial treatment failure (Figure 1). The median age was 30 years [interquartile range (IQR) 27–35] and the median CD4 count was 56 cells/μL (IQR 38–113), see Table 1. KAtex results were negative in 11 (17%), weakly/moderately positive in 17 (27%) and strongly positive (3+) in 35 (36%). KAtex 3+ patients had the lowest CD4 counts, were more likely to have a history of VL at the time of diagnosis and had higher tissue parasite grades, although only the latter association with CD4 count and tissue parasite count reached statistical significance. Twenty (31.7%) patients had parasitologically confirmed initial treatment failure, with a risk of failure of 9% (1/11) with KAtex negative results, 0% (0/17) for KAtex 1+/2+ results and 54% (19/35) for KAtex 3+ result. Compared to KAtex negative patients, KAtex 3+ patients had a statistically significant increased risk of initial treatment failure [odds ratio 11.9 (95% confidence interval (CI) 1.4–103.0); *P*: 0.025], see Table 2.

**Table 1:** Patient characteristics stratified by the level of *Leishmania* urine antigen at the time of VL diagnosis, North Ethiopia (2011–2015).

	**KAtex negative**	**KAtex 1+/2+**	**KAtex 3+**	***P*-value**
Total	11	17	35	
Age, median (IQR)	30 (27–35)	32 (28–40–	30 (27–35)	0.73
Male sex, n (%)	11 (100)	17 (100)	33 (94)	0.44; 1.0
**VL history, n (%)**				
Primary	6 (55)	13 (77)	15 (43)	0.074; 0.78
Relapse	5 (45)	4 (23)	20 (57)	
CD4 count, median (IQR)	66 (48–115)	70 (48–139)	45 (35–60)	0.048
On ART at VL diagnosis, n (%)	3 (30)	8 (61)	25 (73)	0.043; 0.051
Tissue aspirate parasite level				<0.001
1–2, n (%)	6 (55)	5 (29)	4 (11)	
3–5, n (%)	5 (45)	10 (59)	11 (31)	
6+, n (%)	0 (0)	2 (12)	20 (57)	
**VL Treatment**
Antimonials	7 (63)	11 (64)	20 (57)	0.94
Paromomycine	1 (9)	6 (35)	7 (20)	0.26
AmBisome	5 (45)	7 (41)	22 (63)	0.31
Miltefosine	4 (36)	6 (35)	19 (54)	0.22

*ART, antiretroviral treatment; IQR, interquartile range; VL, visceral leishmaniasis*.

**Table 2:** Association between the level of *Leishmania* urine antigen at the time of VL diagnosis and the risk of initial treatment failure, North Ethiopia (2011–2015).

	**Cure; n (column %)**	**Failure; n (column %)**	**Risk of relapse; n (row %)**	**OR (95% CI)**	***P***
**KAtex urine antigen level**					
0	10 (23)	1 (5)	1/11 (9)	1	0.025
1+/2+	17 (40)	0 (0)	0/17 (0)	–^a^	
3+	16 (37)	19 (95)	19/35 (54)	11.9 (1.4–103.0)^b^	
**Tissue aspirate parasite level**					
1+/2+	14 (33)	1 (5)	1/15 (7)	1	<0.001
3+/4+/5+	20 (46)	6 (30)	6/26 (23)	14.5 (0.5–39.9)	
6+	9 (21)	13 (65)	9/22 (43)	20.3 (2.3–178.2)	

*CI, confidence interval; HR, hazard ratio; OR, odds ratio; VL, visceral leishmaniasis*.

a *OR could not be calculated due to the zero value*.

b *The association remained statistically significant after accounting for a history of VL at CD4 count at VL diagnosis (OR 12.0; 95% CI 1.2-115.8; P 0.032)*.

**Figure 2:**
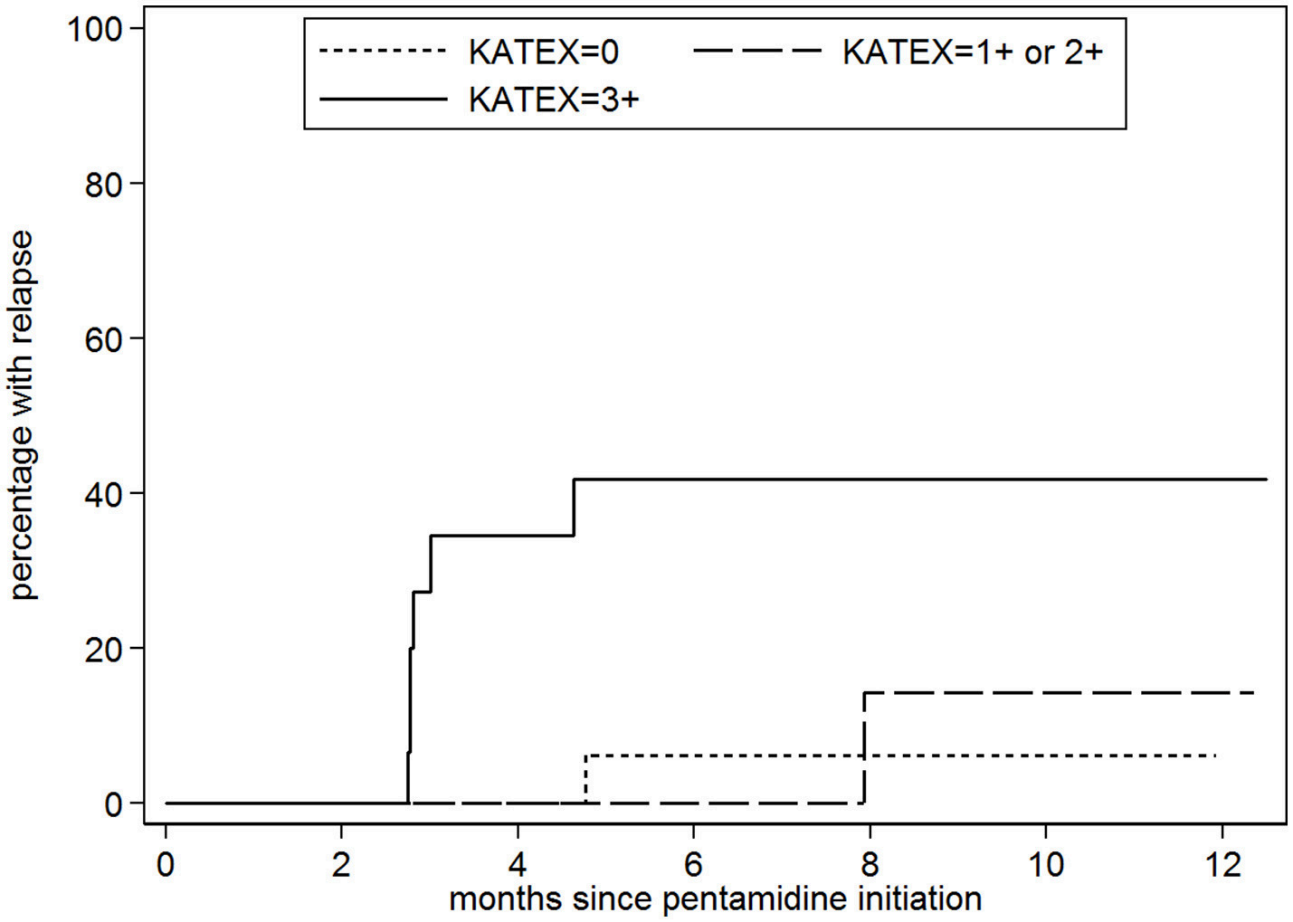
Probability of visceral leishmaniasis (VL) relapse stratified by the level of *Leishmania* urine antigen (KAtex) at the end of treatment (VL cure). KAtex 0: *n* = 19; KAtex 1+/2+: 10; *n* = x; KAtex 3+: *n* = 15.

For the analysis assessing the association between the KAtex result at the time of cure and the risk of subsequent relapse, 44 patients were included (Figure 1). The median age was 31 years (IQR 28–35); all but two were male (95%), see Table 3. Nineteen (43%) were enrolled after a primary VL episode; most (66%; *n* = 29) were on ART at the time VL was diagnosed. The median CD4 count at VL cure was 116 cells/μL (IQR 95–181). When achieving VL cure, KAtex results were negative in 19 (43%), weakly/moderately positive in 10 (23%) and strongly positive (3+) in 15 (34%). KAtex 3+ patients were more likely to have a history of VL, to be on ART at the time of VL diagnosis, and had higher tissue parasite grades at VL diagnosis, although only the latter association with tissue parasite count reached statistical significance. Over the subsequent 12 months, four patients died, seven were lost to follow-up and eight patients relapsed. The predicted 1 year relapse risk was 6% for KAtex negative tests, 14% for KAtex 1+/2+ results, and 42% for KATEX 3+ results [hazard ratio of 2.2 (95% CI 0.1–34.9) for KAtex 1+/2+ and 9.8 (95% CI 1.8–82.1) for KAtex 3+, compared to KAtex negative patients; *P*: 0.03], see Figure 2, Table 4.

**Table 3:** Patient characteristics stratified by the level of *Leishmania* urine antigen at the end of VL treatment (VL cure), North Ethiopia (2011–2015).

	**KAtex negative**	**KAtex 1+/2+**	**KAtex 3+**	***P*-value**
Total	19	10	15	
Age, median (IQR)	29 (28–35)	32 (28–35)	32 (27–40)	0.60
Male sex, n (%)	18 (95)	10 (100)	14 (93)	1.0
**VL history, n (%)**				0.19
Primary	11 (58)	4 (40)	4 (27)	
Relapse	8 (42)	6 (60)	11 (73)	
CD4 count, median (IQR); *n* = 42	119 (95–181)	132 (80–302)	116 (99–134)	0.95
On ART at VL diagnosis, n (%)	11 (58)	5 (50)	13 (87)	0.097
Tissue aspirate parasite level				0.001
1–2, n (%)	8 (42)	2 (20)	0 (0)	
3–5, n (%)	9 (47)	2 (20)	4 (27)	
6+, n (%)	2 (11)	6 (60)	11 (73)	
**VL treatment**				
Antimonials	11 (58)	6 (60)	9 (60)	1.0
Paromomycine	3 (16)	2 (20)	2 (20)	1.0
AmBisome	9 (47)	7 (70)	11 (73)	0.28
Miltefosine	7 (37)	5 (50)	10 (67)	0.22

*ART, antiretroviral treatment; IQR, interquartile range; VL, visceral leishmaniasis*.

**Table 4:** Association between the level of *Leishmania* urine antigen at the time of VL cure and the risk of VL relapse over the subsequent 12 months, North Ethiopia (2011–2015).

	**No relapse; n (column %)**	**Relapse; n (column %)**	**Risk of relapse; n/N (row %)**	**HR (95% CI)**	***P***
**KAtex urine antigen level**					
0	18 (50)	1 (12.5)	1/19 (5)	1	0.026
1+/2+	9 (25)	1 (12.5)	1/10 (10)	2.2 (0.1–34.9)	
3+	9 (25)	6 (75)	6/15 (40)	9.8 (1.8–82.1)	
**Tissue aspirate parasite level**					
1+/2+	10 (28)	0 (0)	0/10 (0)	1^a^	0.025
3+/4+/5+	14 (39)	1 (12)	1/15 (7)	1^a^	
6+	12 (33)	7 (88)	7/19 (37)	11.0 (1.3–89.6)	

*CI, confidence interval; HR, hazard ratio; VL, visceral leishmaniasis*.

a *Due to the zero value in the reference category, these two categories were merged*.

## Discussion

To the best of our knowledge, this is the first study evaluating the value of *Leishmania* antigenuria at the time of VL diagnosis to predict increased risk of initial treatment failure in HIV coinfected patients. Higher levels of *Leishmania* antigen allowed to identify those at highest risk of failure. In addition, higher levels of *Leishmania* antigen in the urine at the time of VL cure were associated with an increased risk of subsequent VL relapse in Ethiopian VL-HIV coinfected patients receiving pentamidine secondary prophylaxis. In Europe, where *L infantum* is prevalent, a positive KAtex test during the post-treatment follow-up of HIV patients has been found predictive of VL relapse (Riera et al., 2004). Equally, detection of parasite DNA in the peripheral blood was also predictive of VL relapse in Europe (Antinori et al., 2007; Bourgeois et al., 2008; Molina et al., 2013; Nicodemo et al., 2013; Bhattacharyya et al., 2014; Cota et al., 2017; Verma et al., 2017). As far as we know, no such studies in HIV patients have been conducted on *L dononavi*.

The level of *Leishmania* antigen in the urine correlated with the level of parasites present in the tissue aspiration, which was also predictive of initial treatment failure. Consequently, this non-invasive test could be used for risk stratification to identify those at higher risk of failure in settings where invasive test such as tissue aspiration are not feasible (e.g., at the decentralized level).

A positive urine antigen test indicates the presence of viable or dead (degraded) parasites. In one European study, parasites could be cultured from the blood of asymptomatic HIV patients with positive urine antigen tests after VL treatment, indicating that viable parasites still remain detectable and in circulation in some patients after treatment (Riera et al., 2004). In *L infantum* endemic areas in France, continuous replication and circulation of the *L infantum* parasite was demonstrated over a period of up to 10 years, both during asymptomatic phases and symptomatic VL relapse episodes. This entity was defined as “active chronic VL” (Bourgeois et al., 2010). In our study, it was impossible to determine whether the positive urine antigen test found at the end of treatment indicates the presence of more live parasites remaining somewhere in the body, or whether it perhaps reflects a higher initial parasite burden which is still being cleared. Of interest is that out of the seven relapse patients with KAtex results available during the trial, six (86%) of them remained antigen positive while on PSP, whereas only 12 (44%) out of 27 patients who remained relapse-free were KAtex positive while on PSP (data not shown).

In our study, there was a positive association between the tissue parasite load at the initial VL diagnosis and both the level of *Leishmania* antigen in the urine at VL diagnosis and at the end of treatment. A high tissue parasite load at VL diagnosis was also associated with relapse. Individuals with a higher initial parasite burden might have higher amounts of residual parasites at the end of treatment, which could be correlated with higher amounts of *Leishmania* urine antigen. On the other hand, this high tissue burden could also be an expression of a pronounced, persistent and more detrimental T-helper 2 cellular response, unable to clear the intracellular parasites.

We previously reported a fair diagnostic accuracy of KAtex for VL diagnosis in VL/HIV co-infection, with a sensitivity of 84% and a specificity of 99% (Vogt et al., 2017). Adding a serological marker could potentially further improve diagnostic accuracy. This could pave the way for a diagnostic work-up combining a serological test with a urine antigen test, with the latter containing prognostic information on who is most likely to fail initial treatment. Given the current limited availability of VL drugs, these patients could potentially be selected for the most effective treatment (e.g., combination therapy). The level of urine antigen at the time of cure could be used before discharge to identify those at highest risk of relapse, who could be targeted for closer medical follow-up or (prolonged) secondary prophylaxis. Development of a dipstick format would further increase field implementation. More sensitive urine antigen tests have recently been developed (Vallur et al., 2015), and their diagnostic and prognostic value in HIV coinfected patients should be evaluated as well.

There are several limitations to this study. As a small exploratory study, findings should be interpreted cautiously and remain to be confirmed in larger, prospective studies. Laboratory results were missing for some. Moreover, our findings on the risk of relapse come from patients undergoing secondary prophylaxis. However, a similar observation was made in an *L infantum* endemic area. Moreover, we do not see a biological reason why our observed association would only apply to patients on secondary prophylaxis. As most of the patients were on antiretroviral therapy at VL diagnosis, or received it early after VL diagnosis, the findings might not be generalizable to settings with limited antiretroviral treatment coverage. It would also have been of interest to collect urine samples during the post-treatment follow-up period and assess the association with subsequent relapse. One of the strengths of the study is that it was nested within a clinical trial, adhering to good clinical and laboratory practices, ensuring that data quality was high.

In conclusion, higher level of *Leishmania* antigen in the urine at the time of VL diagnosis and cure was associated with an increased risk of initial treatment failure and relapse in HIV-patients, respectively. A simple *Leishmania* urine antigen test that can be deployed in resource-limited settings can be used to tailor patient management. Development of a dipstick format would further increase field implementation. Newer urine antigen tests should be evaluated as well.

## Ethics statement

This study was carried out in accordance with the recommendations of the Declaration of Helsinki 2013, the Good Clinical Practice of the WHO, and those of the Ethiopian Food, Medicine and HealthCare Administration and Control Authority (FMHACA) with written informed consent from all subjects. All subjects gave written informed consent in accordance with the Declaration of Helsinki. The protocol was approved by the Ethiopian regulatory authority, the National Research Ethics Review Committee, the University of Gondar Institutional Review Board (IRB), the Ethics Review Board of Médecins sans Frontières, the IRB of the Institute of Tropical Medicine, Antwerp and the Ethics Committee of Antwerp University Hospital.

## Author contributions

JvG and ED conceived the study. BM, TM, HF, YT, EA, RM, KR, FV, and WA contributed in data acquisition. Analysis was done by JvG. Interpretation of data was done by JvG, ED, and KR. JvG drafted the first draft of the manuscript. ED, BM, TM, HF, YT, EA, RM, KR, FV, and WA commented on the first draft of the manuscript. All authors read and approved the final manuscript.

### Conflict of interest statement

The authors declare that the research was conducted in the absence of any commercial or financial relationships that could be construed as a potential conflict of interest.
